# A preliminary study of KAT2A on cGAS-related immunity in inflammation amplification of systemic lupus erythematosus

**DOI:** 10.1038/s41419-021-04323-1

**Published:** 2021-10-30

**Authors:** Youzhou Tang, Xinyu Li, Yafang Wei, Yongchao Sun, Yeyi Yang, Xianming Zhang, Zhihao Gao, Jishi Liu, Quan Zhuang

**Affiliations:** 1 Department of Nephropathy and Rheumatology, the 3rd Xiangya Hospital, Central South University, Changsha, China; 2 Xiangya School of Medicine, Central South University, Changsha, China; 3 Transplantation Center, the 3rd Xiangya Hospital, Central South University, Changsha, China; 4 Research Center of National Health Ministry on Transplantation Medicine, Changsha, China

**Keywords:** Autoimmunity, Lupus nephritis

## Abstract

Previous studies demonstrated that cGAS pathway is related to the inflammation amplification in a variety of autoimmune diseases. Lysine acetyltransferase family (KATs) can regulate the nuclear transcription or cytoplasmic activation of cGAS through different mechanisms. However, its role and related immunity patterns in systemic lupus erythematosus (SLE) have not been explored. In this study, RNA-seq and scRNA-seq profiling were performed for peripheral blood mononuclear cells (PBMCs) from patients with SLE. R packages were used for bioinformatic analysis. Cell culture, RT-PCR, western blotting, immunofluorescence, immunohistochemistry, and ELISA were used to explore gene expression in vitro or clinical specimens. Plasmid transfection and mass spectrometry were used to detect protein modifications. Eight acetyltransferase and deacetylase family members with significantly differential expression in SLE were found. Among them, KAT2A was abnormally upregulated and positively correlated with disease activity index. Further, KAT2A-cGAS pathway was aberrantly expressed in specific immune cell subsets in SLE. In vitro studies showed KAT2A modulated cGAS through increasing expression and post-translational modification. Our research provides novel insights for accurately positioning specific immune-cell subgroups in which KAT2A-cGAS reaction mainly works and KAT2A regulation patterns.

## Introduction

Systemic lupus erythematosus (SLE) is an autoimmune disease with complex etiology. Uncontrolled inflammation amplification in SLE leads to multiple organs and tissue damage, including skin, kidney, heart, and etc. [1]. However, the underlying mechanism of this inflammation amplification is elusive. Cyclic GMP-AMP synthase (cGAS) senses cytoplasmic DNA and activates downstream signals, including second messenger cyclic GMP-AMP (cGAMP), stimulator of interferon genes (STING), tank binding kinase 1 (TBK1), and interferon regulatory factor 3 (IRF3), which then translocate into the nucleus and regulates type I interferon (IFN-I) transcription [2, 3]. Aberrant activation of cGAS stimulated by self-DNA has been enrolled in multiple autoimmune diseases, especially SLE [4–6]. The inflammation augmentation and immune-cell activation of SLE depend on the cGAS-STING pathway in patient cells and murine model [7, 8]. Conversely, the regulation of cGAS activation in specific immune cell is still unclear.

cGAS is regulated by various posttranslational modifications at its C-terminal catalytic domain. Histone acetylating modification is one of the main forms of epigenetic modification. Cytoplasmic molecular lysine (K) acetylation is an important post-translational modification way for functional protein activation [9]. There are two types of enzymes regulating these processes: [1] lysine acetyltransferases (KATs) translocate acetyl from acetyl-CoA into ε-amino acid side chains; [2] lysine deacetylases (KDACs) reverse this acetylation process. KAT2A (lysine acetyltransferase, GCN5) belongs to KATs family, which exerts its effectiveness through modifying histone and other functional molecules, such as CDC6, cyclin A, PLK4, and etc. [10–12]. Hence, KAT2A participates in many biological activities, such as DNA and chromatin modification, cell cycle progression, etc. Nonetheless, whether KAT2A could regulate inflammatory molecules in SLE has not been investigated yet.

In this study, we identified KAT2A from KATs by public database and uncovered that KAT2A not only was related to SLE activity, but affected cGAS activation. So, we speculated that KAT2A regulated and activated cGAS activation in inflammation amplification of SLE. Through single-cell transcriptomics sequencing (scRNA-seq) analysis, we inferred that simultaneous upregulation of KAT2A and cGAS in SLE had been involved in the abnormal activation of multiple immune-cell subpopulations, including dendritic cells (DCs), monocytes, T and B lymphocytes. Our findings suggest that KAT2A may regulate cGAS activation and subsequently affect the inflammation amplification in immune cells and will provide new clues for SLE targeted therapy.

## Methods

### Patient cohort and data preparation

The RNA sequencing (RNA-seq) cohort of the study contained 198 adult patients with SLE from the Gene Expression Omnibus (GEO, available at: https://www.ncbi.nlm.nih.gov/geo/) database (GSE138458) [13]. The single-cell transcriptomics sequencing (scRNA-seq) data was obtained from GSE135779, including adult peripheral blood mononuclear cells (PBMCs) from six healthy controls and seven patients with SLE [14]. According to the median systemic lupus erythematosus disease activity index (SLEDAI), seven SLE scRNA-seq samples were divided into SLEDAI_high and SLEDAI_low groups. According to the anti-double strains (ds)DNA antibodies, seven patients were divided into dsDNA(+) and dsDNA(−) groups.

From January 2020 to May 2021, patients with SLE were enrolled from the Department of Nephropathy and Rheumatology of the 3rd Xiangya Hospital, Central South University. All the samples were collected when the patients were firstly diagnosed as SLE and without any previous treatment. All procedures in this study followed the 1975 Helsinki Declaration revised in 2008. The study was reviewed and approved by the Ethics Committee in the 3rd Xiangya Hospital, Central South University (IRB No. 21029). All patients participated in this study, satisfied SLE classification standard of the American College of Rheumatology [15]. All patients were excluded from current acute infection, chronic HIV, HBV, HCV, and syphilis. The patients’ specific information, including SLEDAI, age, sex, clinical symptoms etc., was collected and stated in Supplementary Table 1. Renal tissues were collected from patients with lupus nephritis (LN) or without renal autoimmune disease (kidney surgery).

### RT-PCR and western blot

PBMCs were extracted from the five healthy controls and 13 patients with SLE (5 ml blood each sample) by Ficoll-Hypaque (GE Healthcare, Uppsala, Sweden) density-gradient centrifugation. After total RNA was extracted by TRIzol reagent (Thermo, 15596026), reverse transcription (RT) was performed using mRNA RT reagent kit (Beijing cwbiotech, CW2569). RT reaction system included dNTP Mix (4ul), Primer Mix (2 μl), RNA Template (7 μl), 5 × RT Buffer (4 μl), DTT (2 μl), HiFiScript (1 μl). SYBR products were used for each gene expression quantification in real-time PCR measurement. Primers were designed by primer5 software, and specific primers were listed in Supplementary Table 2. Relative RNA was calculated using 2^−ΔΔCt^ normalized to β-actin.

RIPA (Shanghai Beyotime biotechnology, P0013B) was used for protein extraction. After centrifugation (12000 rpm, 15 min), BCA (ACE biotechnology, AL006-01) was used to measure protein concentration. Protein samples were loaded on 10% gradient gel and run electrophoresis after which were transformed into nitrocellulose membrane. Primary antibodies were listed in Supplementary Table 3. Images were analyzed using quantity one software. For each protein expression, protein band density/GAPDH band density was used to represent specific protein quantitative measurement.

### Immunohistochemistry and immunofluorescence

Renal tissues were obtained from patients with LN (IV + V) by biopsy and without renal autoimmune disease by kidney surgery. Tissue immunohistochemistry and PBMC cellular immunofluorescence were performed to detect cGAS and KAT2A proteins. Paraffin-embedded renal tissues were used for immunohistochemistry. 5−10% goat serum was used for blocking, then samples were incubated with primary cGAS antibody overnight (4 °C). After washing for three times, samples were incubated for 30 min with biotin-binding secondary antibody. The liquid was dropped on samples which then were been washed for three times with PBS. HRP-Streptavidin diluted with PBS was added and incubated for 10 min at 37 °C. Samples were washed for three times with PBS, AEC was used for visualization.

PBMCs were fixed and permeabilized in 0.3% Triton for 30 min. Non-specific sites were blocked with goat serum for 60 min at 37 °C. Samples were incubated with primary antibodies (KAT2A, cGAS) overnight at 4 °C. Secondary goat anti-mouse IgG (H + L) (1:200, Proteintech) were incubated with samples for 90 min at 37 °C. DAPI (Wellbio) was used for nucleus staining for 10 min. After washing for three times with PBS, samples were observed under fluorescence microscope (×400). Positive signals were shown as green fluorescence for cGAS, red for KAT2A, blue for DAPI.

### Cytokine detection (ELISA)

Supernatants were collected after cell culture, centrifugated for 15 min at 1000 × *g*. IFN-I ELISA kit (FANKEW F10665-A) was used for standard and test samples incubation, washing, reaction, HRP coloration. Curve Expert software was used to set up standard curve, OD value was used to calculate each sample’s IFN-I concentration.

### Plasmid transfection

HEK293 (293A) cells were cultured in DMEM with 10% FBS and cell passage was done in proportion 1:2−1:3. 0.25% trypsin was used for cell digestion. Collect cells after centrifugation (1000 rpm, 5 min) and inoculate cells (5 × 10^6^ per pore) into a 10 cm plate overnight. Lip2000 (Invitrogen 2028090) was used for plasmid transfection. Add Lip2000 with corresponding plasmids (pcDNA3.1-cGAS-3xFlag, pcDNA3.1-KAT2A-Myc, HonorGene HG-HO138441 & HG-HO 021078) and DMEM into the plate and incubate 6 h under room temperature, then change culture medium into DMEM with 10% FBS. Collect cells after 48 h (5%CO2 and 37 °C). Plasmid information was shown in Supplementary Fig. 1. Cell lines are obtained from the agent (HonorGene, China) and tested for mycoplasma contamination before use.

### Cell culture and KAT2A inhibition experiment in vitro

PBMCs obtained from six individuals were cultured in RPMI-1640 medium containing 10% fetal bovine serum (FBS). PBMCs from one individual were divided into two groups: cultured with 0.8 mmol/l KAT2A inhibitor or equal volume phosphate-buffered saline (PBS) for 24 h. We applied CPTH2 (C14H14ClN3S) to inhibit KAT2A, which is a histone acetyltransferase inhibitor modulating the KAT2A network [16].

### Cell culture and KAT2A overexpression experiment in vitro

THP-1 cells were cultured in RPMI-1640 medium containing 10% fetal bovine serum (FBS). Cells were divided into four groups: (1) transfected with KAT2A plasmid (pcDNA3.1-KAT2A-Myc); (2) transfected with KAT2A plasmid (pcDNA3.1-KAT2A-Myc) and dsDNA stimulant (e.coli DNA plasmid); (3) transfected with empty plasmid; (4) transfected with empty plasmid and dsDNA stimulant (e.coli DNA). Cell lines are obtained from the agent (HonorGene, China) and tested for mycoplasma contamination before use.

### Mass spectrometry for cGAS lysine loci determination

HEK293 cells were transfected either with FLAG-cGAS and Myc-KAT5 plasmids or only FLAG-cGAS plasmids for 24 h. Protein was extracted from lysed cells, immunoprecipitated with anti-FLAG agarose beads, and tested by mass spectrometry (Bruker timsTOF Pro) for acetylation and other post-transcription modification sites, which was supported by Xiamen University.

### Bioinformatic analysis

All analyses were performed with R version 4.0.2 (https://www.r-project.org/) and the corresponding packages. The limma algorithm was used to identify differentially expressed genes (DEGs) between the two groups. GSEA and GO were applied to determine the functional or pathway enrichment within a given gene set. The scRNA-seq was mainly analyzed by Seurat package.

### Statistical analysis

Statistical calculations were performed using GraphPad Prism version 9.0 (GraphPad Software Inc., La Jolla, CA, USA) or SPSS version 23.0 (SPSS, Inc., Chicago, IL, USA). Analysis of Student’s t-test (two-sided) was used to analyze the level of significant difference between the groups, unequal variances with Welch’s t-test. Pearson analysis was performed to identify correlations. All comparisons were perceived to have significant differences when *p* < 0.05. *, **, *** and **** means *p* < 0.05, *p* < 0.01, *p* < 0.001 and *p* < 0.0001, respectively. Definition of ‘center values’ as mean and definition of error bars as SD in the figures.

## Results

### cGAS-STING pathway components were hyper expressed in SLE

**Fig. 1:**
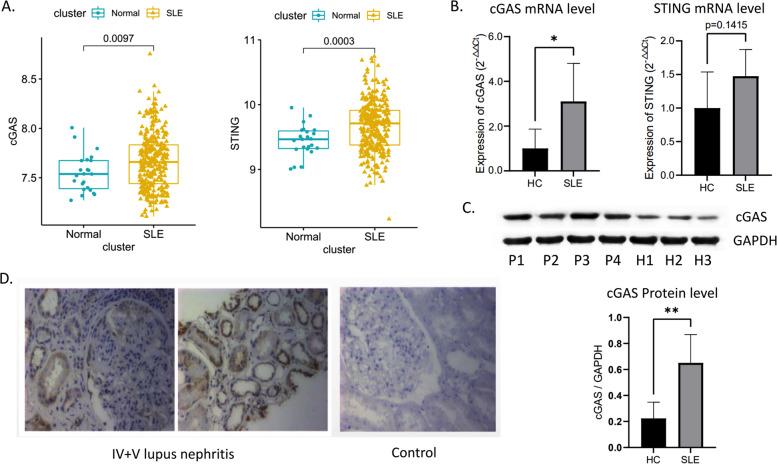
Expresssion levels of cGAS-STING pathway in SLE. Differential expression analysis of cGAS and STING in GSE138458 cohort (**A**); RT-PCR results of cGAS and STING in clinical samples (**B**); western blot results of cGAS in clinical samples. The gray value of “cGAS/GADPH” was used to measure the protein level of cGAS (**C**); immunohistochemistry staining of cGAS in renal tissues (**D**). *, **, *** and **** means *p* < 0.05, *p* < 0.01, *p* < 0.001 and *p* < 0.0001, respectively.

In GEO cohorts, we observed the expression levels of cGAS and STING were increased in SLE compared to healthy controls (Fig. 1A). In clinical specimens, PBMCs from patients with SLE showed a significant elevation of cGAS at both mRNA and protein levels; there was a rising trend of STING mRNA, but not a significant difference (*P* > 0.05) (Fig. 1B, C). Compared to normal renal tissues, an upregulation of cGAS in the renal tissues from LN patients was also observed by immunohistochemistry staining (Fig. 1D).

### Upregulation of cGAS caused inflammation amplification in SLE

**Fig. 2:**
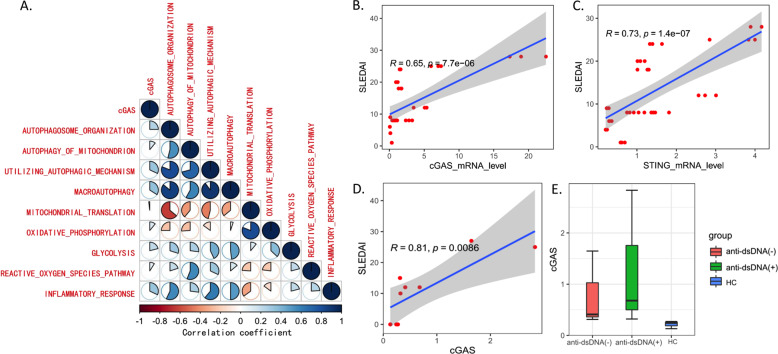
Relationship between cGAS-STING pathway and inflammation in SLE. The heatmap shows the correlation coefficient of ssGSEA enrichment scores among various pathways and *p* < 0.05 was satisfied between any two pathways (**A**); correlation analysis of cGAS-STING mRNA levels with SLEDAI (**B**, **C**); correlation analysis of cGAS protein level with SLEDAI (**D**); protein level of cGAS in groups with different clinical characteristics (**E**).

To explore the correlation between upregulated cGAS expression and inflammation amplification of SLE, we divided the GEO cohorts into two groups based on the median expression of cGAS gene. The DEGs between two groups (*p* < 0.05) were obtained and enriched by GSEA method: the inflammatory response and antigen presentation, as well as IFN-Iα response, were highly kindled in the group with high cGAS expression (Supplementary Fig. 2A–C), which was accompanied by the metabolic reprogramming and autophagy activation (Supplementary Fig. 2D–I). Combined analysis of correlation and ssGSEA showed that inflammation, glycolysis, autophagy, and cGAS could have a positive impact on each other (Fig. 2A). In clinical samples, we verified the significant correlation between cGAS-STING pathway and disease activity indices (SLEDAI) (Fig. 2B–D). Furthermore, there was a rising trend of cGAS protein level in anti-dsDNA(+) patients compared to anti-dsDNA(−) ones and healthy controls, but not a significant difference (Fig. 2E).

### Aberrant expression of KAT2A was closely related to cGAS activation in SLE

**Fig. 3:**
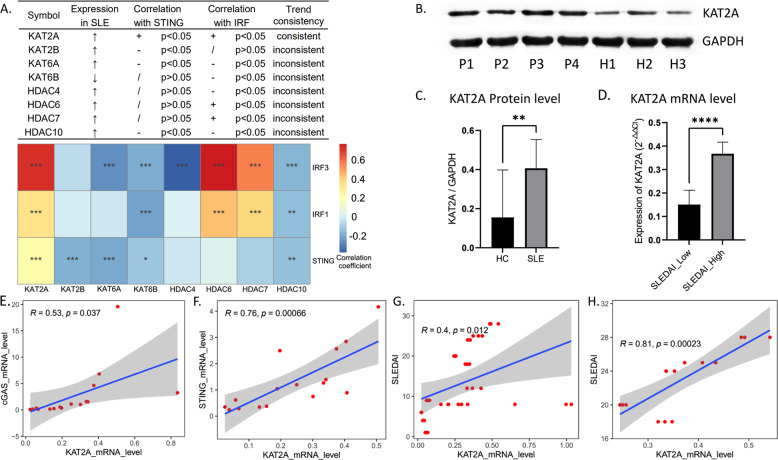
Expression level of KAT2A and its relationship with cGAS-STING pathway. Correlation analysis of 8 acetyltransferase and deacetylase family members with STING and IRFs (**A**); western blot results of KAT2A in clinical samples (**B**); the gray value of “KAT2A/GADPH” was used to measure the protein level of KAT2A (**C**); RT-PCR results of KAT2A in SLE group with different degrees of disease activity index (**D**); Pearson correlation analysis of KAT2A and cGAS pathway expression (**E**, **F**); Pearson correlation analysis of KAT2A expression and SLEDAI in all patients with SLE (**G**); Pearson correlation analysis of KAT2A expression and SLEDAI in patients with SLEDAI above 15 (**H**). *, **, *** and **** means *p* < 0.05, *p* < 0.01, *p* < 0.001 and *p* < 0.0001, respectively.

Given the complex mechanism of cGAS related inflammatory responses, we explored the correlation between acetyltransferase and deacetylase family members and cGAS activation. Eight members with significantly differential expression in SLE were found (Supplementary Fig. 13). By screening, KAT2A was abnormally upregulated in SLE and had a positive correlation with cGAS downstream pathways in GEO cohorts (Fig. 3A). Clinical specimens were collected to further verify that the mRNA and protein levels of KAT2A showed an aberrant elevation in patients with SLE and high SLEDAI group (Fig. 3B–D). Furthermore, KAT2A was significantly correlated with cGAS-STING signaling at mRNA level and disease activity indices (SLEDAI) (Fig. 3E–G), especially in high SLEDAI group (Fig. 3H).

### Aberrant expression of KAT2A and cGAS was associated with specific immune-cell subsets in SLE

**Fig. 4:**
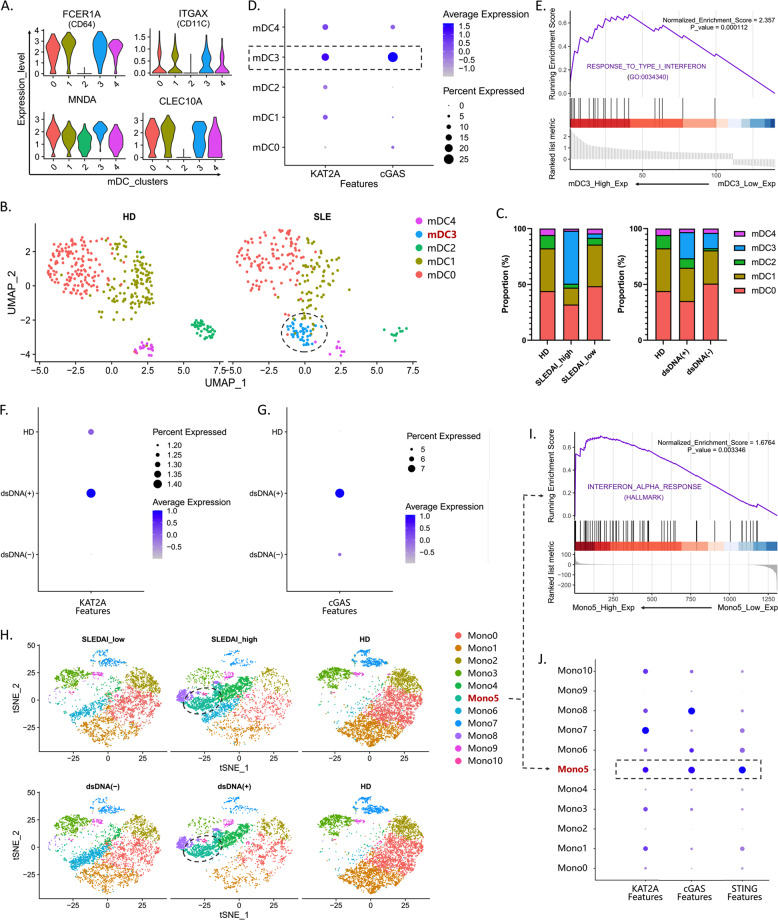
Expression KAT2A and cGAS in T/B lymphocytes from different groups. The violin plot shows the markers used to define mDC (**A**); UMAP plots represent the five clusters of mDCs from 13 individual (7 SLE and 6HD), in which cluster 3 (mDC3) was specific in SLE group (**B**); composition of mDC clusters in groups with different clinical characteristics (**C**); expression level of KAT2A and cGAS in each cluster. The color depth indicates the expression level, and the size of the dot indicates the proportion of cells expressing this gene (**D**); GSEA analysis of specific high expression genes in mDC3. Normalized enrichment score (NES) > 0 means the IFN-1 pathway is upregulated in mDC3 (**E**); expression KAT2A and cGAS in monocytes from different groups. The color depth indicates the expression level and the size of the dot indicates the proportion of cells expressing this gene (**F**, **G**); t-SNE plots represent the 11 clusters of monocytes from samples with different clinical characteristics (**H**); GSEA analysis of specific high expression genes in cluster 5 (Mono5). Normalized enrichment score (NES) > 0 means the IFN-1 pathway is upregulated in Mono5 (**I**); expression level of KAT2A and cGAS in each cluster (**J**).

**Fig. 5:**
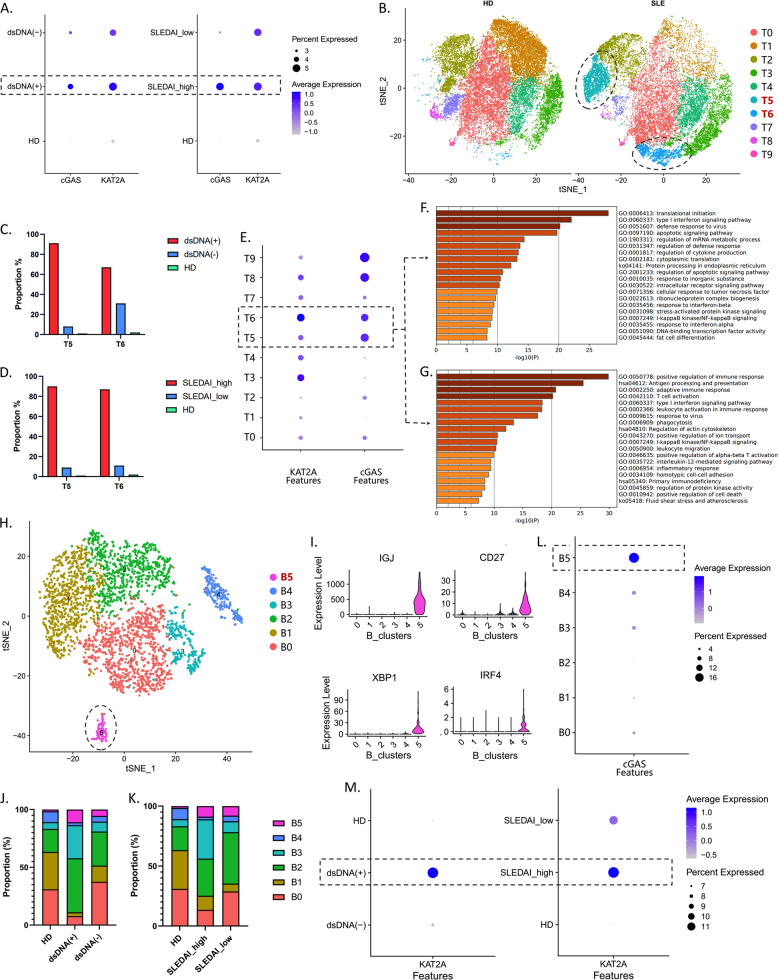
Expression KAT2A and cGAS in myeloid cells from different groups. The color depth indicates the expression level and the size of the dot indicates the proportion of cells expressing this gene (**A**); t-SNE plots represent the 10 clusters of T cells from 13 individual (7 SLE and 6HD), in which clusters 5 and 6 (T5 and T6) were specific in SLE group (**B**); the proportion of T5 and T6 clusters in groups with different clinical characteristics (**C**, **D**); expression level of KAT2A and cGAS in each cluster (**E**); GO analysis of specific high expression genes in T5 (**F**) and T6 (**G**); t-SNE plots represent the six clusters of B cells (**H**); the violin plot shows the markers used to define effector B cells, in which cluster 5 (B5) was identified (**I**); composition of B clusters in groups with different clinical characteristics (**J**, **K**); expression level of cGAS in each cluster (**L**); expression of KAT2A in B cells from different groups (**M**).

The PBMCs were extracted from SLE patients and healthy controls, and cell clusters were analyzed based on the stepwise approach via scRNA-seq. Canonical markers were applied to define clusters. We used gene expression of “FCGR1A”, “CLEC10A”, “ITGAX”, “CD1C”, and “MNDA” to define myeloid dendritic cells (mDC) (Fig. 4A). The mDCs were further clustered into five subsets, in which mDC3 was only appeared in SLE group compared to healthy controls (Fig. 4B), and its proportion was higher in the SLEDAI_high and dsDNA(+) groups than that of SLEDAI_low and dsDNA(−) ones (Fig. 4C). Besides, mDC3 showed an aberrant expression of KAT2A and cGAS and an upregulation of response to IFN-I (Fig. 4D, E). The expression of “CD68”, “FCGR3A”, “CD14” and “MNDA” was used to define monocytes (Mono). We found that monocytes had higher expression of KAT2A and cGAS in dsDNA(+) group (Fig. 4F, G). Monocytes were clustered into 11 subsets, in which Mono5 was identified to be a special subset in SLEDAI_high and dsDNA(+) groups (Fig. 4H). Similarly, higher expressions of KAT2A, cGAS, and STING were found in this Mono5 subset, as well as response to IFN-I (Fig. 4I, J). SLEDAI_high and dsDNA(+) groups also had a high expression of KAT2A and cGAS in T cells (Fig. 5A). 10 subsets were clustered in T cells, and the proportions of T5 and T6 subsets were much higher in the SLEDAI_high and dsDNA(+) groups than that of SLEDAI_low, dsDNA(−) ones, and healthy controls (Fig. 5B–D). Besides, T5 and T6 subsets showed a high expression of KAT2A and cGAS (Fig. 5E), whose functional enrichment showed a high correlation with IFN-I signaling pathway and the activation of multiple immune responses (Fig. 5F, G). In B cells, 6 subsets were clustered in B cells (Fig. 5H). The expression of “IGJ”, “CD27”, “XBP1” and “IRF4” was used to define plasma cells (B5) (Fig. 5I). The proportion of B5 subset was relatively higher in the SLEDAI_high and dsDNA(+) groups than that of SLEDAI_low, dsDNA(−) ones, and healthy controls (Fig. 5J, K), and this B5 subset highly expressed cGAS (Fig. 5L). Besides, SLEDAI_high and dsDNA(+) groups had a high expression of KAT2A in B cells (Fig. 5M).

### Potential regulation of KAT2A on cGAS in SLE

**Fig. 6:**
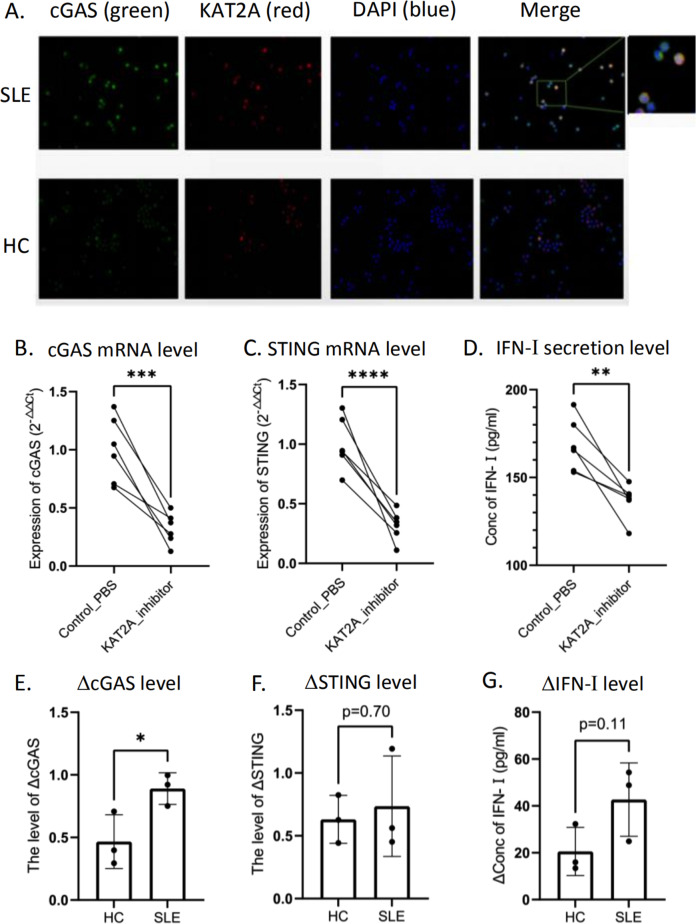
Potential regulation of KAT2A on cGAS in SLE. Positive signals were shown as green fluorescence for cGAS, red for KAT2A, blue for DAPI (**A**); the obtained PBMCs from SLE group and healthy donors co-cultured with PBS or KAT2A inhibitor respectively. The expression level of cGAS pathway was detected by RT-PCR after co-culture for 24 h (**B**, **C**); the concentration of IFN-1 in the supernatant was detected by ELISA after co-culture (**D**); difference of detection indices after different treatment (co-cultured with PBS or KAT2A inhibitor) in the same sample (**E**−**G**). Samples from healthy donors or patients have inconsistent levels of this difference. *, **, *** and **** means *p* < 0.05, *p* < 0.01, *p* < 0.001 and *p* < 0.0001, respectively.

We next preliminarily explored how KAT2A regulated the expression and activation of cGAS. Immunofluorescence showed that KAT2A and cGAS were co-located in both cytoplasm and nucleus in PBMCs from patients with SLE (Fig. 6A). Under KAT2A inhibition, the transcriptional level of cGAS was inhibited and the activation of downstream interferon pathways was limited (Fig. 6B–D). This inhibitory effect was more obvious in PBMCs of SLE group than that of healthy controls (Fig. 6E–G). In THP-1 cells, we also investigated the effect of KAT2A overexpression on monocyte interferon response by transfecting overexpression plasmid. We found that overexpression of KAT2A in monocyte lines could not significantly increase interferon secretion (Supplementary Fig. 4), which suggested that the role of KAT2A is different in various immune cells.

Since KAT2A not only exerts its role in the nucleus, cGAS activation may also be regulated by post-translational modification. We compared the cGAS protein mass spectra of 293A cells that were transfected with KAT2A & cGAS plasmid versus cells transfected with cGAS plasmid only. When KAT2A was transfected, the acetylation state of cGAS changed at multiple sites. Three new acetylation sites in samples that were transfected with KAT2A & cGAS plasmid were found in Table 1 and Supplementary Fig. 5.

**Table 1:** Protein mass spectra of cGAS from different samples.

Samples	Sequence	Modifications
Transfected with KAT2A & cGAS plasmid	AVLEKLK	1xAcetyl [K5]
	DDISTAAGMVK	1xOxidation [M9]
	FSSYHVK	1xAcetyl [K7]
	GAPMDPTESPAAPEAALPK	1xOxidation [M4]
	MLSKFR	1xAcetyl [K4] 1xOxidation [M1]
Transfected with cGAS plasmid only	ASARNARGAPMDPTESPAAPEAALPKAGK	1xAcetyl [K26]
	ASEAGATAPK	1xAcetyl [K10]
	ATGARAKK	2xAcetyl [K7; K8]
	KSGSRQKK	3xAcetyl [K1; K7; K8]
	RGGSPAVTLLISEK	1xAcetyl [K14]

We compared the cGAS protein mass spectra of samples that were transfected with KAT2A & cGAS plasmid versus samples transfected with cGAS plasmid only. When KAT2A was transfected, there were three new acetylation sites in different peptides.

## Discussion

cGAS has been studied to have the crucial role in mediating self-DNA accumulated diseases, especially SLE [5, 17]. In the light of its importance in inflammation, previous studies have focused on its downstream effects. However, there was a rare study on the regulatory mechanism of cGAS activation. Recently, acetylation modification has been valued for its regulatory effect on cGAS activation. For one, the transcriptional level of cGAS pathways can be regulated by histone acetylation modification; for another, different lysine loci acetylation composition acts diversely on cGAS DNA sensation ability and may lead to various effects on cGAS activation. For instance, K384/K394/K414 acetylation inhibits cGAS activation while K47/K52/K62/K83 acetylation activates cGAS [8, 18], which indicates different lysine sites acetylation combination would possibly lead to different effect on cGAS activation. Based on these, we firstly screened out acetyltransferase KAT2A from acetylation and deacetylation enzymes by bioinformatic analysis from the existing SLE public database and explored its potential regulation effect on cGAS activation and the inflammation amplification of SLE.

Approximately 400 non-histone proteins have been identified to be regulated by KAT2A/2B [12]. KAT2A/2B not only acetylates these proteins but also regulates either negatively or positively their cellular biological and pathological function, such as translocation and phosphorylation. In this study, we speculated and obtained clues that KAT2A might promote the inflammation amplification of SLE via regulation on cGAS: [1] the upregulated KAT2A co-localizes with cGAS in PBMCs and positively related with cGAS and disease activity in SLE; [2] KAT2A inhibition down-regulated cGAS, STING at mRNA level, as well as IFN-I expression in SLE. KAT2A might have an impact on active SLE by targeting cGAS transcription.

**Fig. 7:**
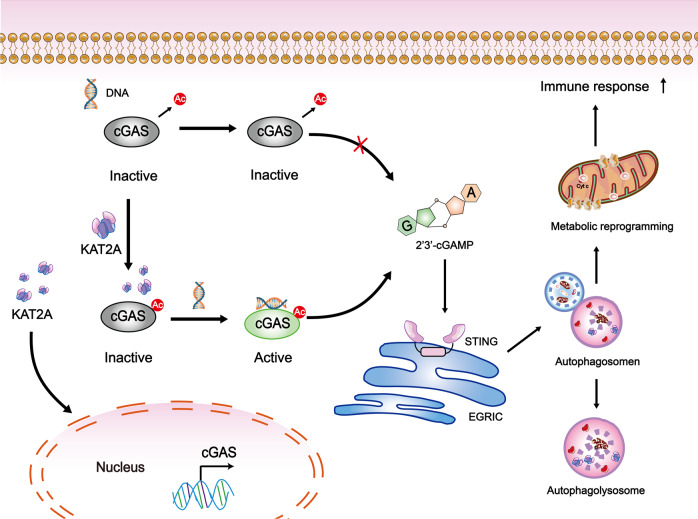
The mechanical explanation of the findings in our study. KAT2A may have multiple roles in the nucleus and cytoplasm to regulate cGAS, such as acetylation and deacetylation, transcription regulation, etc. The activated cGAS-STING pathway may induce inflammation amplification by affecting autophagy and metabolic reprogramming. Multiple abnormal immune-cell subgroups devoted to SLE inflammation enlargement.

As an acetylation enzyme, KAT2A has recently been found not only to exert its effect on histone but also regulate multiple biological and pathological processes [19–22]. In these studies, KAT2A combines with other different functional protein on its particularly structural domains, and these combinations could play a role in cell migration, autophagosome formation, and apoptosis, etc. In our study, we found that KAT2A directly affected the total acetylation level of cGAS and targeted three lysine loci for KAT2A acetylation by mass spectrometry, which was in accordance with a previous study that acetylation could block cGAS activity while deacetylation could increase cGAS activation under DNA stimulation [8]. Since we sorted out three special cGAS lysine sites for KAT2A regulation, further studies could focus more on KAT2A’s enzymatic function on cGAS functional domain. Together with these results, we infer that KAT2A may have multiple roles in the nucleus and cytoplasm to regulate cGAS (Fig. 7), which is worthy to explore more on acetylation and deacetylation, transcription regulation, etc.

Multiple abnormal immune-cell subgroups devoted to SLE inflammation enlargement. Few studies have been launched on KAT2A and cGAS in specific immune cells of patients with SLE. We did find some interesting results based on cells with different KAT2A expression levels. Although inhibition of KAT2A in PBMCs derived from lupus patients can inhibit interferon secretion, overexpression of KAT2A in monocyte lines could not significantly increase interferon secretion based on our data. Possible explanations for this result are as follows: (1) The activation of interferon signal is complex and may be regulated by various factors. Independent overexpression of KAT2A may not have the competence to fully activate interferon signal. (2) Functional differences between immune cells should be considered. The results observed in PBMCs may be the result of the interaction of multiple immune cells. At least our results suggest that monocytes may not be the main regulatory object of KAT2A. This further indicates the necessity of studying the mechanism of KAT2A on cGAS-STING-IFN pathway in different cell subsets.

We additionally identified and analyzed multiple immune-cell subpopulations in T, B lymphocytes, dendritic cells (DC), and monocytes in PBMCs from patients via scRNA-seq and found there was an obvious imbalance of these subsets under SLE condition and a high correlation with KAT2A, cGAS, and STING. Meaningfully, besides the lymphocytes which are the main effector cells of SLE in the traditional standpoints, innate immune cells, such as DCs and monocytes showed a tight correlation with the SLE progression [23–25]. DCs usually located with T cells in the inflammatory sites of LN patients and expressed surface molecules in a more mature state to trigger T cell activation [23]. cGAS-STING signaling pathway exerts irreplaceable influence on DCs’ recognition for accumulated DNA [7, 26], which implied cGAS signaling pathway was important for DC activation and antigen presentation Hence, it is worthy to elucidate precise cGAS activation process in precise DC subgroups enrolled in SLE inflammation enlargement. Recently, single-cell analysis of human mononuclear phagocytes focused on several immune-cell subtypes and selected out specific DC subpopulation for SLE specialty. Both plasmacytoid DC and conventional DC subpopulations have been investigated to not only have imbalance in SLE but also aberrant upregulated interferon-stimulated genes (ISG), which is the downstream effect of cGAS signaling pathway [14, 27]. This is in accordance with our speculation and results. Consequently, mDC subgroups shown in our analysis, which have simultaneously upregulated KAT2A and cGAS are undoubtedly worthy for further investigation. In addition, high expression of cGAS-STING in effector B cells was observed in our study. Recent studies have shown that STING can regulate BCR signal through PI3K, so as to regulate humoral immunity. It is suggested that cGAS-STING also contributed to the adaptive immune response in lupus [28]. This may give us meaningful clues to develop novel and effective therapeutic targets for patients with SLE.

This study has several limitations. The number of samples is not large enough, so that some results are not significant. The main results focused on PBMCs from clinical samples. Although the landscape of immune cell subsets has been studied by single-cell sequencing, the conclusion has not been verified in vitro. Further studies should be carried out to explore the precise mechanism of KAT2A regulating cGAS through gene interference/knockout experiments and confirm the localization of immune cell subsets.

In conclusion, regulation of cGAS by KAT2A may be an important molecular mechanism for SLE inflammation amplification. Accurately positioning specific immune-cell subgroups in which KAT2A-cGAS reaction mainly works and KAT2A regulation patterns are two crucial orientations for SLE targeted therapy exploration.

## Supplementary information

Supplementary materials

## Data Availability

Publicly available datasets and permitted data access were analyzed in this study. These data can be found here: Gene Expression Omnibus (GEO, available at: https://www.ncbi.nlm.nih.gov/geo/) database (GSE138458), and the single-cell transcriptomics sequencing (scRNA-seq) data was obtained from GSE135779.

## References

[1] Lou H, Wojciak-Stothard B, Ruseva MM, Cook HT, Kelleher P, Pickering MC (2020). Autoantibody-dependent amplification of inflammation in SLE. Cell Death Dis.

[2] Sun L, Wu J, Du F, Chen X, Chen ZJ (2013). Cyclic GMP-AMP synthase is a cytosolic DNA sensor that activates the type I interferon pathway. Science.

[3] Wu J, Sun L, Chen X, Du F, Shi H, Chen C (2013). Cyclic GMP-AMP is an endogenous second messenger in innate immune signaling by cytosolic DNA. Science.

[4] Zhang X, Bai XC, Chen ZJ (2020). Structures and mechanisms in the cGAS-STING innate immunity pathway. Immunity.

[5] Gao D, Li T, Li XD, Chen X, Li QZ, Wight-Carter M (2015). Activation of cyclic GMP-AMP synthase by self-DNA causes autoimmune diseases. Proc Natl Acad Sci USA.

[6] Kumar V (2019). A STING to inflammation and autoimmunity. J Leukoc Biol.

[7] Thim-Uam A, Prabakaran T, Tansakul M, Makjaroen J, Wongkongkathep P, Chantaravisoot N (2020). STING mediates Lupus via the activation of conventional dendritic cell maturation and plasmacytoid dendritic cell differentiation. iScience.

[8] Dai J, Huang YJ, He X, Zhao M, Wang X, Liu ZS (2019). Acetylation blocks cGAS activity and inhibits self-DNA-induced autoimmunity. Cell.

[9] Narita T, Weinert BT, Choudhary C (2019). Functions and mechanisms of non-histone protein acetylation. Nat Rev Mol Cell Biol.

[10] Paolinelli R, Mendoza-Maldonado R, Cereseto A, Giacca M (2009). Acetylation by GCN5 regulates CDC6 phosphorylation in the S phase of the cell cycle. Nat Struct Mol Biol.

[11] Orpinell M, Fournier M, Riss A, Nagy Z, Krebs AR, Frontini M (2010). The ATAC acetyl transferase complex controls mitotic progression by targeting non-histone substrates. EMBO J.

[12] Fournier M, Orpinell M, Grauffel C, Scheer E, Garnier JM, Ye T (2016). KAT2A/KAT2B-targeted acetylome reveals a role for PLK4 acetylation in preventing centrosome amplification. Nat Commun.

[13] Guthridge JM, Lu R, Tran LT, Arriens C, Aberle T, Kamp S (2020). Adults with systemic lupus exhibit distinct molecular phenotypes in a cross-sectional study. EClinicalMedicine.

[14] Nehar-Belaid D, Hong S, Marches R, Chen G, Bolisetty M, Baisch J (2020). Mapping systemic lupus erythematosus heterogeneity at the single-cell level. Nat Immunol.

[15] Hochberg MC (1997). Updating the American College of Rheumatology revised criteria for the classification of systemic lupus erythematosus. Arthritis Rheum.

[16] Chimenti F, Bizzarri B, Maccioni E, Secci D, Bolasco A, Chimenti P (2009). A novel histone acetyltransferase inhibitor modulating Gcn5 network: cyclopentylidene-[4-(4’-chlorophenyl)thiazol-2-yl)hydrazone. J Med Chem.

[17] Kato Y, Park J, Takamatsu H, Konaka H, Aoki W, Aburaya S (2018). Apoptosis-derived membrane vesicles drive the cGAS-STING pathway and enhance type I IFN production in systemic lupus erythematosus. Ann Rheum Dis.

[18] Song ZM, Lin H, Yi XM, Guo W, Hu MM, Shu HB (2020). KAT5 acetylates cGAS to promote innate immune response to DNA virus. Proc Natl Acad Sci USA.

[19] Ouyang C, Mu J, Lu Q, Li J, Zhu H, Wang Q (2020). Autophagic degradation of KAT2A/GCN5 promotes directional migration of vascular smooth muscle cells by reducing TUBA/α-tubulin acetylation. Autophagy.

[20] Sandoz J, Nagy Z, Catez P, Caliskan G, Geny S, Renaud JB (2019). Functional interplay between TFIIH and KAT2A regulates higher-order chromatin structure and class II gene expression. Nat Commun.

[21] Helmlinger D, Papai G, Devys D, Tora L (2021). What do the structures of GCN5-containing complexes teach us about their function?. Biochim Biophys Gene Regul Mech.

[22] Wang Y, Guo YR, Liu K, Yin Z, Liu R, Xia Y (2017). KAT2A coupled with the α-KGDH complex acts as a histone H3 succinyltransferase. Nature.

[23] Kassianos AJ, Wang X, Sampangi S, Muczynski K, Healy H, Wilkinson R (2013). Increased tubulointerstitial recruitment of human CD141(hi) CLEC9A(+) and CD1c(+) myeloid dendritic cell subsets in renal fibrosis and chronic kidney disease. Am J Physiol Ren Physiol.

[24] Soni C, Perez OA, Voss WN, Pucella JN, Serpas L, Mehl J (2020). Plasmacytoid dendritic cells and type I interferon promote extrafollicular B cell responses to extracellular self-DNA. Immunity.

[25] Menon M, Blair PA, Isenberg DA, Mauri C (2016). A regulatory feedback between plasmacytoid dendritic cells and regulatory B cells is aberrant in systemic Lupus Erythematosus. Immunity.

[26] Kim JT, Liu Y, Kulkarni RP, Lee KK, Dai B, Lovely G, et al. Dendritic cell-targeted lentiviral vector immunization uses pseudotransduction and DNA-mediated STING and cGAS activation. Sci Immunol. 2017;2:eaal1329. 10.1126/sciimmunol.aal1329 PMC5845465 28733470

[27] Dutertre CA, Becht E, Irac SE, Khalilnezhad A, Narang V, Khalilnezhad S (2019). Single-cell analysis of human mononuclear phagocytes reveals subset-defining markers and identifies circulating inflammatory dendritic cells. Immunity.

[28] Jing Y, Dai X, Yang L, Kang D, Jiang P, Li N (2020). STING couples with PI3K to regulate actin reorganization during BCR activation. Sci Adv.

