# Supplementary material for: A preliminary study of KAT2A on cGAS-related immunity in inflammation amplification of systemic lupus erythematosus
Source: Cell Death Dis. 2021 Oct 30;12(11):1036. doi: 10.1038/s41419-021-04323-1 (PMC8557211; doi:10.1038/s41419-021-04323-1)
Supplement: Supplementary file 1 — Supplementary materials [file 41419_2021_4323_MOESM1_ESM.pdf]

Supplementary materials

Supplementary tables

Supplementary Table 1. Patient information of clinical samples.

ID	Name	Group	SLEDAI	Age	Sex	Anti-dsDNA
732841	CJJ	SLE	4	64	female	negative
722038	FFJ	SLE	8	46	female	negative
741911	GZ	SLE	8	16	male	negative
254120	HY	SLE	28	39	female	negative
706639	HSY	SLE	1	56	male	negative
747121	LP	SLE	25	31	female	positive
493550	SHY	SLE	18	41	female	negative
744523	QXH	SLE	6	30	female	negative
171893	TP	SLE	9	59	female	negative
484362	WQF	SLE	24	52	female	positive
700662	WXY	SLE	20	43	female	negative
744412	WYQ	SLE	12	50	female	positive
716152	XCX	SLE	8	58	female	negative
749786	YJ	SLE	23	23	female	negative
742810	LLF	SLE	12	39	female	negative
755694	HDQ	SLE	19	24	female	negative
442350	WX	SLE	12	34	female	negative
419717	ZP	SLE	21	19	female	positive

Supplementary Table 2. Primers used in our study.

Target	Forward Primer	Reverse Primer
β-actin	ACCCTGAAGTACCCCATCGAG	AGCACAGCCTGGATAGCAAC
cGAS	GCTACTATGAGCACGTGAAGATTT	TGAATTCTGGGGACTTCCAGT
STING	GAGGTTACTGTGGGCAGCTT	TGATGAGGAGCTCAGGCTCT
KAT2A	CAGGGTGTGCTGAACTTTGTG	TCCAGTAGTTAAGGCAGAGCAA

Supplementary Table 3. Primary antibodies used in this research.

Target	Antibody company	Antibody Source	Dilution Ratio
KAT2A	ABclonal A2224	Rabbit	0.180555556
cGAS	ABclonal A8335	Rabbit	0.388888889
GAPDH	Proteintech 10494-1-AP	Rabbit	3.513888889

Supplementary figures

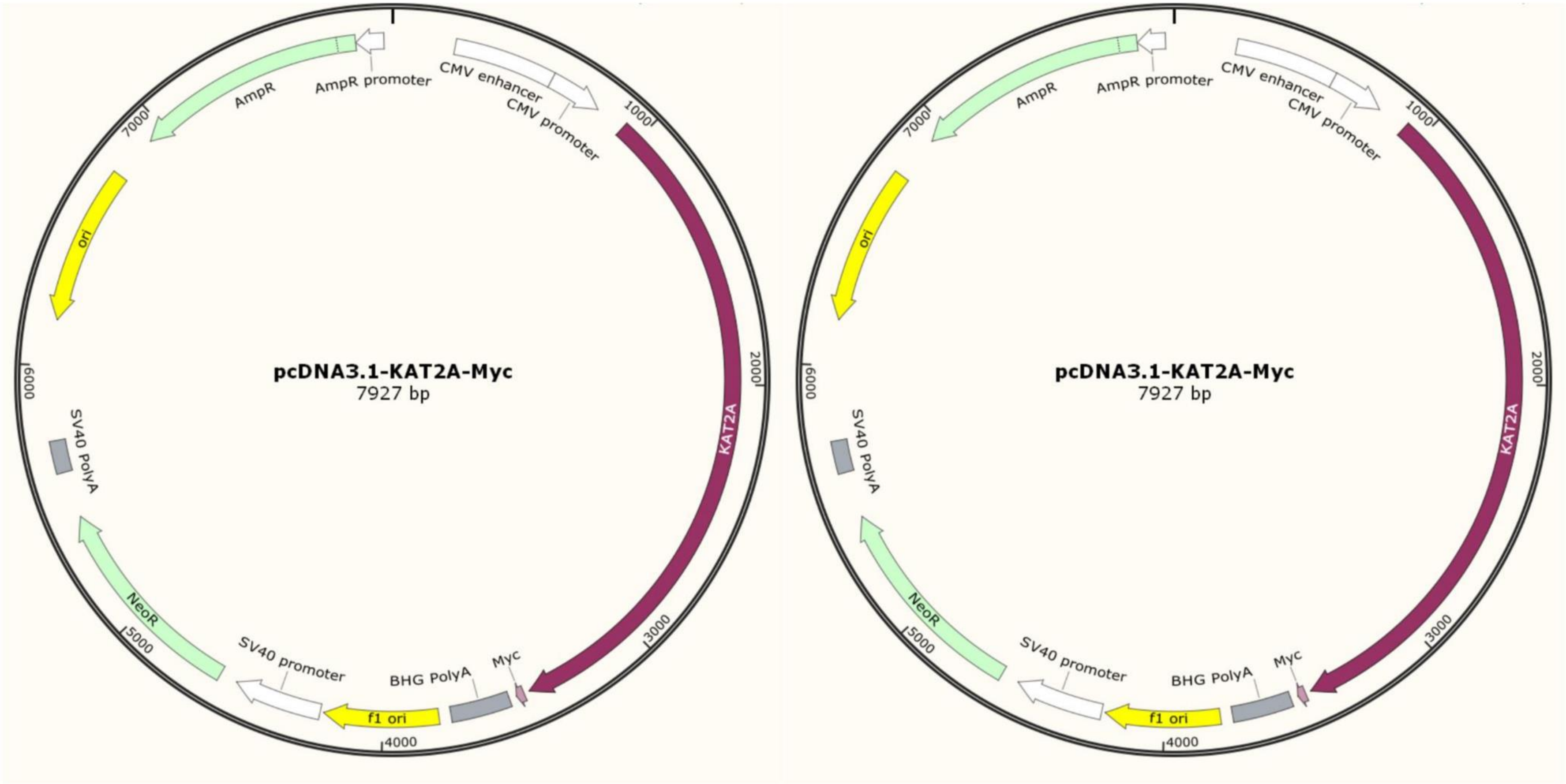

Supplementary Figure 1. The plasmid maps show the details of plasmid construction.

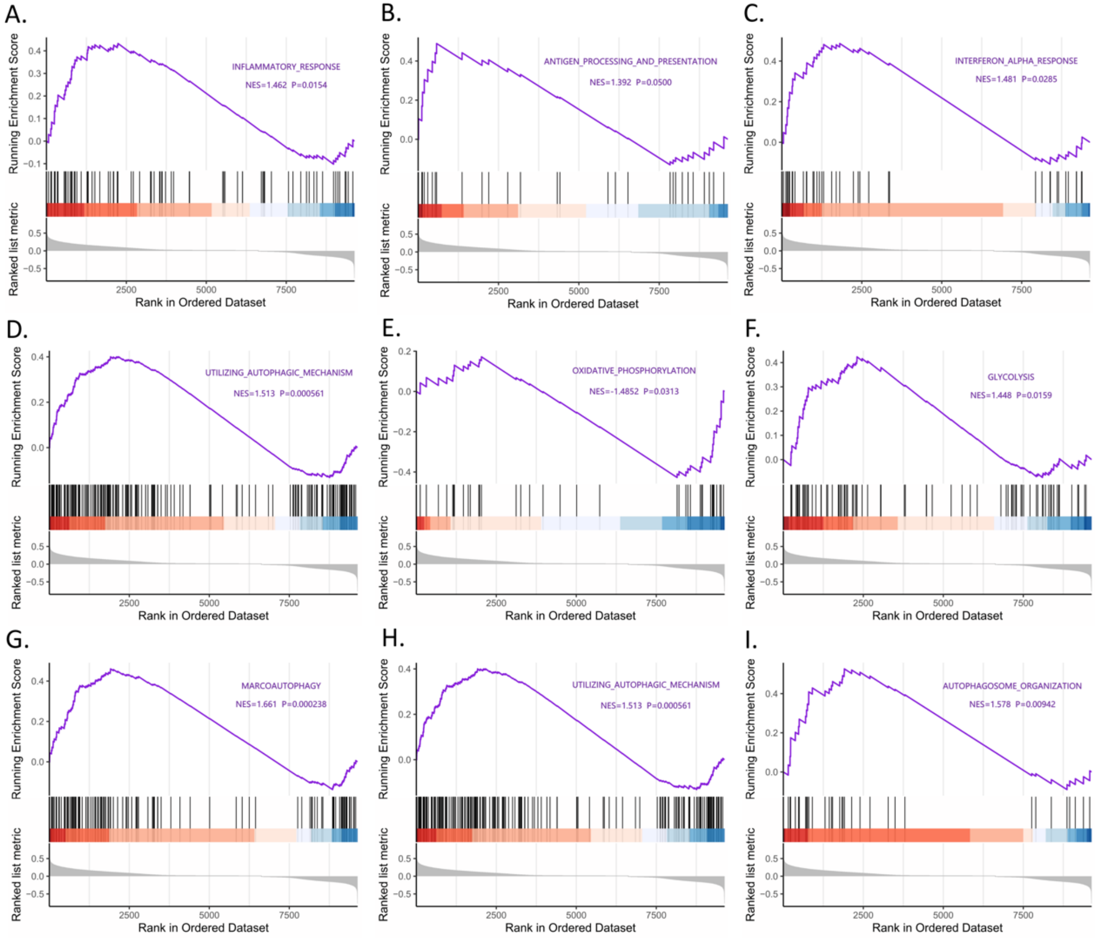

Supplementary Figure 2. GSEA analysis of DEGs between the two groups. Normalized enrichment score (NES) > 0 means the pathway is enriched in the SLE group's up-regulated genes.

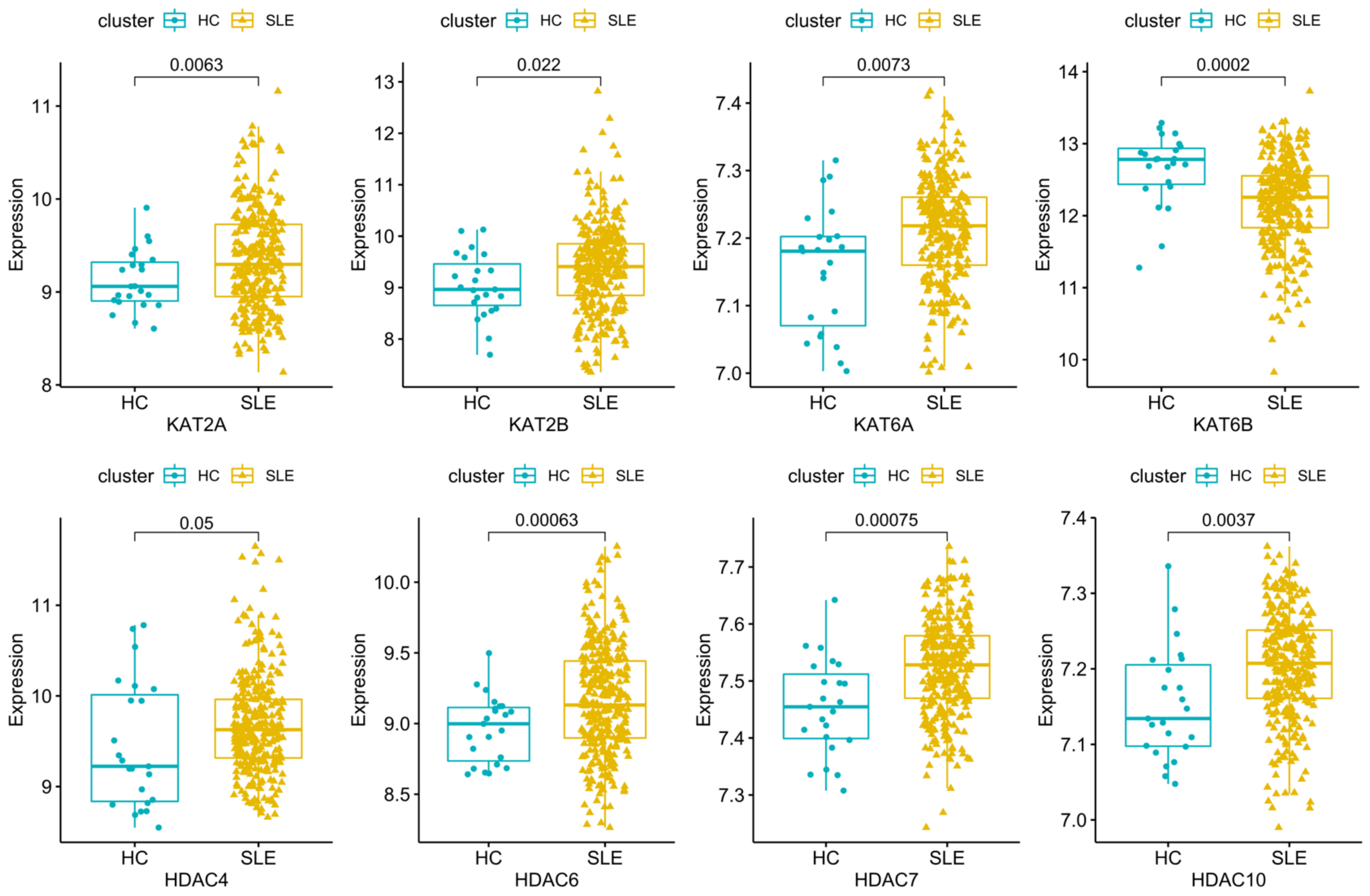

Supplementary Figure 3. Eight acetyltransferase and deacetylase family members with significant differential expression in SLE have been found in GSE138458 cohort.

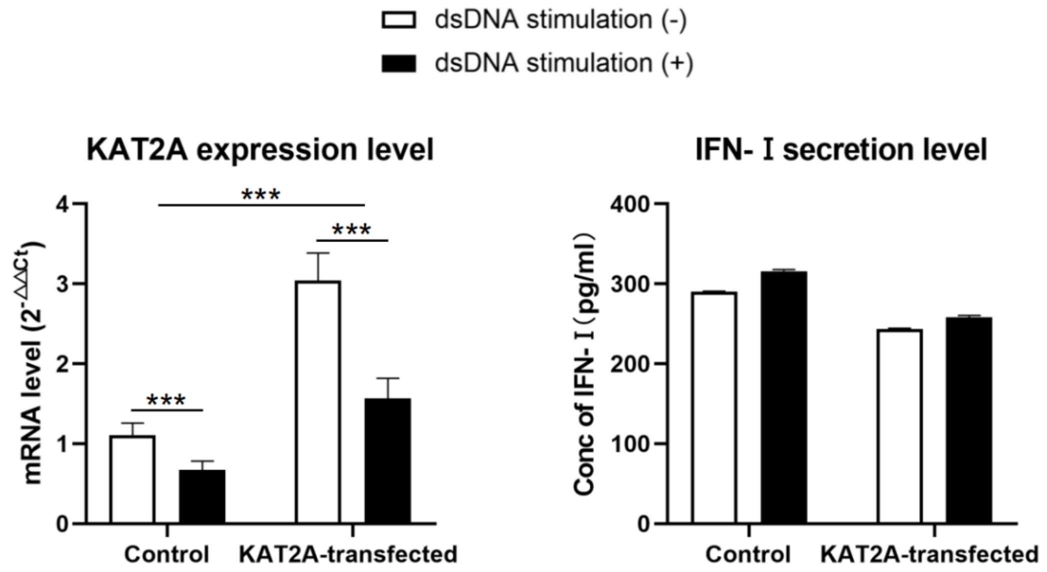

Supplementary Figure 4. THP-1 cells were divided into four groups: 1) KAT2A overexpression plasmid (pcDNA3.1-KAT2A-Myc) was transfected; 2) KAT2A overexpression plasmid (pcDNA3.1-KAT2A-Myc) and dsDNA stimulant (e.coli DNA) were transfected; 3) empty plasmid was transfected; 4) empty plasmid and dsDNA stimulant (e.coli DNA) were transfected. We found that overexpression of KAT2A in THP-1 cells could not significantly increase interferon secretion.

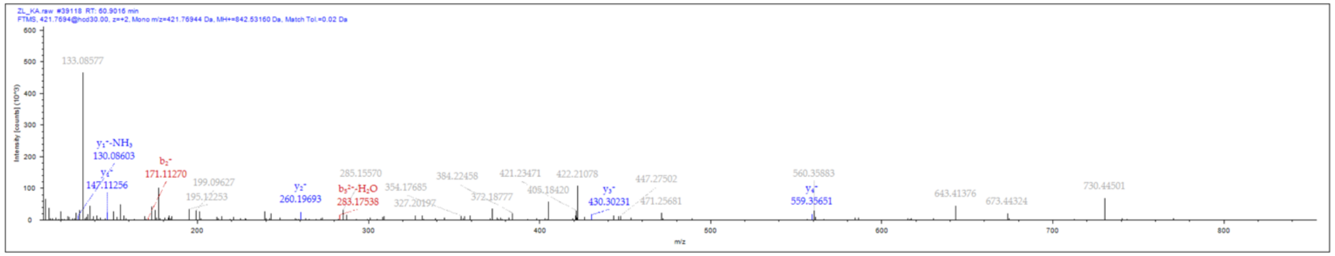

(1) Mass spectra results of peptide "AVLEKLK"

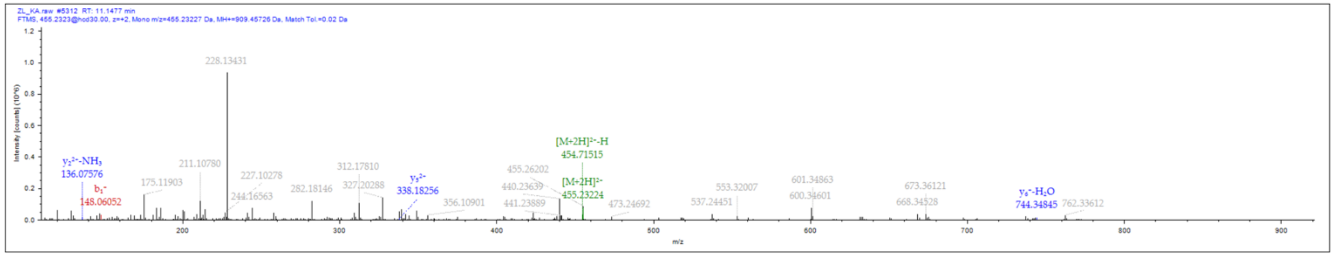

(2) Mass spectra results of peptide "FSSYHVK"

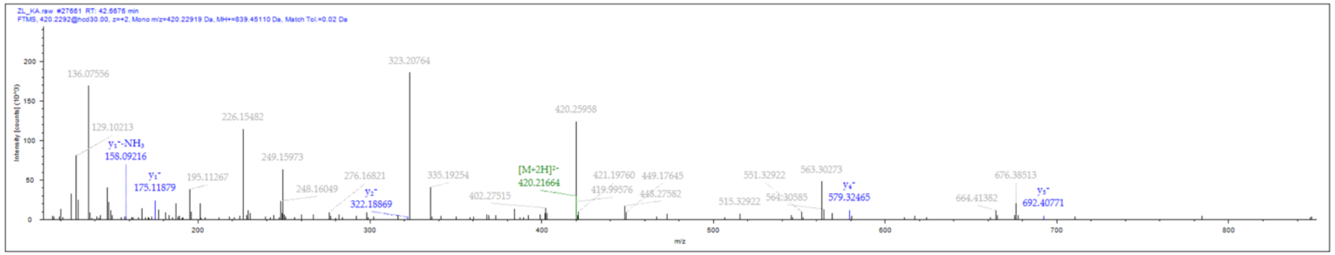

(3) Mass spectra results of peptide "MLSKFR"

Supplementary Figure 5. Three new acetylation sites in samples that transfected with KAT2A & cGAS plasmid were found. Mass spectra plots show these sites in different peptides.
